# Secretase promotes AD progression: simultaneously cleave Notch and APP

**DOI:** 10.3389/fnagi.2024.1445470

**Published:** 2024-11-20

**Authors:** Ke-Fan Yang, Jing-Yi Zhang, Mei Feng, Kuo Yao, Yue-Yang Liu, Ming-Sheng Zhou, Hui Jia

**Affiliations:** ^1^Department of Pathology and Pathophysiology, School of Basic Medical Sciences, Shenyang Medical College, Shenyang, China; ^2^Department of Pharmacy, General Hospital of Northern Theater Command, Shenyang, Liaoning, China; ^3^Science and Experimental Research Center of Shenyang Medical College, Shenyang, Liaoning, China; ^4^School of Traditional Chinese Medicine, Shenyang Medical College, Shenyang, Liaoning, China

**Keywords:** α, β, γ, δ, ε, η-secretase, Notch, cancer, AD, APP

## Abstract

Alzheimer’s disease (AD) involves complex pathological mechanisms. Secretases include membrane protein extracellular structural domain proteases and intramembrane proteases that cleave the topology to type I or type II. Secretases can effectively regulate the activation of Notch and amyloid precursor protein (APP), key factors in the progression of AD and cancer. This article systematically summarizes the intracellular localization, cleavage sites and products, and biological functions of six subtypes of secretases (α-secretase, β-secretase, γ-secretase, δ-secretase, ε-secretase, and η-secretase), and for the first time, elucidates the commonalities and differences between these subtypes of secretases. We found that each subtype of secretase primarily cleaves APP and Notch as substrates, regulating Aβ levels through APP cleavage to impact the progression of AD, while also cleaving Notch receptors to affect cancer progression. Finally, we review the chemical structures, indications, and research stages of various secretase inhibitors, emphasizing the promising development of secretase inhibitors in the fields of cancer and AD.

## 1 Introduction

Secretases are membrane protein extracellular domain hydrolases responsible for cleaving the topology structure (type I or type II). γ-secretase is an intramembrane protease which cleave type I membrane proteins at cleavage sites deep within the hydrophobic regions that are localized deep within the hydrophobic region of the alpha helix of the membrane protein substrates, distinctly different from the other ones that cleave loops that are outside of the hydrophobic core of the lipid bilayer. There are six common subtypes of secretases: α-secretase, β-secretase, γ-secretase, δ-secretase, ε-secretase, and η-secretase. Secretases are involved in the secretion process of intracellular protein substances. The small molecular fragments produced are released into the extracellular environment through secretion after synthesis is completed inside the cell, exerting their biological functions (Pham et al., 2017).

**FIGURE 1:**
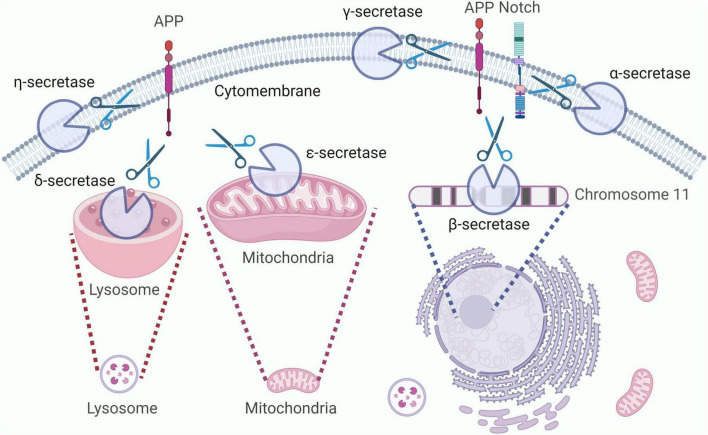
Localization and cleavage of various subtypes of secretase. α, γ, and η-secretases are mainly localized on the cell membrane, while β-secretase is located on chromosome 11, δ-secretase is located in lysosomes, and ε-secretase is located on the inner mitochondrial membrane. All subtypes of secretases can cleave APP, with α, β, and γ-secretases also capable of cleaving the Notch receptor. APP intracellular domain.

α-secretase can break down proteins into small peptide molecules and amino acids, located on the surface of the cell membrane, its main function is to cleave specific sites of APP (Lichtenthaler, 2011). β-secretase, also known as beta-site amyloid precursor protein cleaving enzyme 1 (BACE1), is located on chromosome 11 and is responsible for cleaving APP to generate soluble extracellular domain APP-beta and its C-terminal fragment CTF-beta. γ- secretase is an intracellular membrane protein complex located mainly in the endoplasmic reticulum and Golgi apparatus membranes of cells (Wolfe, 2009). It is involved in the cleavage and degradation of proteins on the cell membrane, mainly involving the degradation of two types of substrates, APP and Notch receptors, and plays an important role in the progression of breast cancer (BC) and AD (Hur, 2022). δ-secretase is the only known mammalian asparagine endopeptidase (AEP), located in the endo-lysosomal system, responsible for cleaving APP and promoting the production of amyloid-beta (Aβ) (Zhang Z. et al., 2020). ε-secretase can catalyze the hydrolysis of succinyl-CoA in the tricarboxylic acid cycle, mainly located on the inner mitochondrial membrane. Epsilon-secretase can also cleave APP and generate different lengths of the C-terminal APP intracellular domain (AICD) (Chang and Suh, 2010). η-secretase is located on the cell membrane, participating in the APP processing pathway, generating carboxy-terminal fragment-η (CTF-η or η-CTF) and Aη-α peptide (Ward et al., 2017). The localization of each subtype of secretase inside the cell, as shown in Figure 1.

The various subtypes of secretases both distinguish and closely relate to each other. They all act on APP as a substrate, but the cleavage sites and products are different. α-secretase acts on the Aβ sequence in APP, cleaving at position 687 to release the soluble fragment s-APPα, while also generating the carboxy-terminal C83 fragment with 83 amino acids (Lichtenthaler, 2011). β-secretase cleaves at the 670th methionine and 671st aspartic acid residues of APP, generating the N-terminus of Aβ. The cleavage sites of γ-secretase and ε-secretase are located on the C99 side chain of APP, producing different lengths of AICD (Chang and Suh, 2010). The Notch intracellular domain (NICD) derived from the cleavage of APP by ε-secretase can also regulate p53 (Checler et al., 2007). More than 90 type I transmembrane proteins have been reported to be cleaved by γ-secretase, but not all of these substrates are directly related to physiological processes. γ-secretase has a high degree of substrate specificity, and it tends to recognize specific amino acid sequences and structural features of proteins (Beel and Sanders, 2008). More research is needed to determine the specific roles of these substrates in cellular physiology and disease. The δ-secretase cleaves APP at the N585 site, generating the 586–695 fragment of APP, and can also trigger apoptosis and tumor metastasis by hydrolyzing phosphatidylinositol on the cell membrane (Zhang Z. et al., 2020). η-secretase cleavage mainly occurs at amino acid sequence 504–505 of the 695 segment of APP, releasing extracellular domain fragments, which can generate high molecular weight carboxyl-terminal fragments of APP (Willem et al., 2015). The cleavage sites and hydrolysis products of common substrates for each subtype of secretase are shown in Table 1.

**TABLE 1:** Shear the sites and products of each subtype of secretase.

Subtypes	Shear site	Product
α-secretase	687th point of the APP	C83 fragment
β-secretase	The 670th cysteine and the 671st aspartic acid residues in the APP	C99 fragment
γ-secretase	On the side chains of C83 and C99 in the APP	AICD fragment
δ-secretase	The N585th point of the APP	CTF-δ fragment
ε-secretase	C99 sidechain on the APP	AICD fragment
η-secretase	Amino acids at position 504–505 in segment 695 of the APP	CTF-η fragment

APP, amyloid precursor protein; AICD, APP intracellular domain; CTF-δ, carboxy-terminal fragment-δ; CTF-η, carboxy-terminal fragment-η.

## 2 Biological functions of secretase

The α-secretase plays an important biological role in cell development and organism function. α-secretase, α-disintegrin and metalloproteinase-10 (ADAM10), can recognize and cleave specific substrate protein APP, and its binding with Tspan15 antibody can form a complex. The ADAM10-Tspan15 complex regulates cell adhesion and mutual recognition, which is beneficial for cell development (Lipper et al., 2023). In addition, α-secretase can affect the development of the cardiovascular system, central nervous system, immune system, intestines, kidneys, synapses, endothelium, epidermis, and hair. Recent studies have shown that inhibiting the activity of α-secretase can lead to inactivation of the Notch pathway, and mice completely lacking α-secretase will die around 10 days of embryonic development. This study reveals that α-secretase affects organism function by mediating the shedding of the extracellular domain of E-cadherin (Rosenbaum and Saftig, 2024).

β-secretase (BACE) includes two homologous forms, namely BACE1 and BACE2. They share about 68% homology but play different biological functions. BACE1 plays a role in retinal lesions, nerve impulses, and organism aging. BACE1 is involved in the homeostasis of retinal pigment epithelium. Low expression of BACE1 can lead to significant retinal pathological changes in mice. Overexpression of BACE1 can prevent retinal degeneration and loss of visual function (Qi et al., 2023). BACE1 is also beneficial for the development of nerve fiber myelin sheaths, regulating axon targeting and cochlear synapse formation. Mice with BACE1 defects have three characteristics: first, abnormal development and impaired function of myelin sheath cells, affecting the speed of nerve impulse conduction. Second, abnormal axon growth and positioning, hindering nerve impulse and neural network formation. Third, abnormal cochlear synapse connections leading to decreased hearing and perception (Dierich et al., 2019). BACE1 can affect stem cell function during the aging process of the body. Research has found that increased activity of BACE1 in the nervous system can negatively regulate neural stem cells by cleaving the corresponding substrate APP. Furthermore, BACE1 can damage the stem cell microenvironment by cleaving Aβ or cleavage-related vascular molecules (Bao and Shen, 2023). The difference is that BACE2 acts as a vascular protective protein in brain blood vessels. In brain endothelial cells, increased BACE2 activity can enhance the vascular protective function of endothelial nitric oxide synthase, including vasodilation, inhibition of platelet aggregation, and prevention of vascular inflammation (He et al., 2023).

**FIGURE 2:**
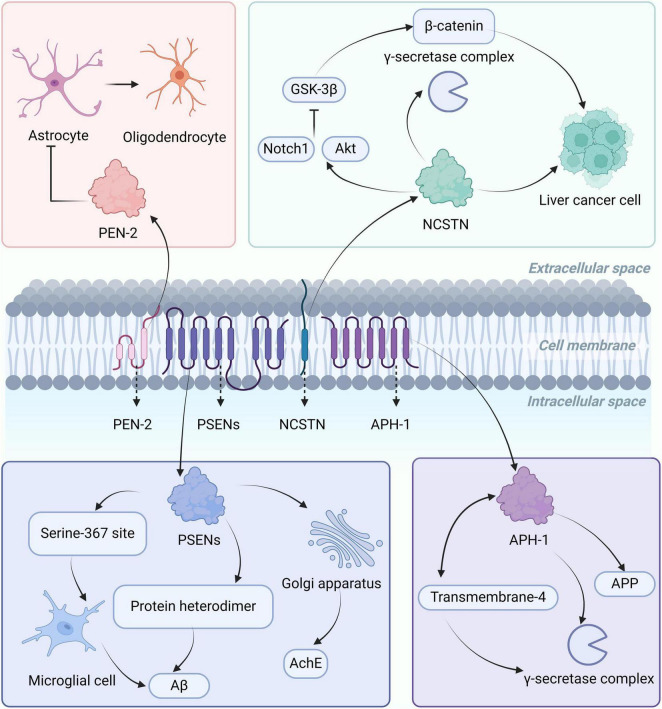
Localization, structure, and biological functions of the γ-secretase complex. The γ-secretase complex is mainly located on the cell membrane and consists of PSENs, NCSTN, APH-1, and PEN-2. PSENs can form heterodimers or regulate astrocytes through phosphorylation at serine 367, affecting the levels of Aβ, and influencing the glycosylation of AchE in the Golgi region. NCSTN can regulate the activity of the γ-secretase complex, activate the Notch1 and AKT signaling pathways, inhibit GSK-3β activity, leading to β-catenin accumulation, and promote the growth of liver cancer cells. APH-1 is involved in the production of APP and interacts with the fourth transmembrane-4 structure region of presenilin, promoting the formation of the γ-secretase complex. PEN-2 regulates the differentiation of oligodendrocyte precursor cells into astrocytes. AChE, acetylcholinesterase; APP, amyloid precursor protein; Aβ, amyloid-beta; PSEN, presenilins; NCSTN, nicastrin; APH-1, anterior pharynx defective protein-1; PEN-2, presenilin enhancer-2.

γ-secretase is a transmembrane protein complex located on the cell membrane, consisting of four key subunits, including presenilins (PSENs), nicastrin (NCSTN), anterior pharynx defective protein-1 (APH-1), and presenilin enhancer-2 (PEN-2) (Feng et al., 2024), each subunit has different biological functions, as shown in Figure 2. PSENs can form heterodimers in the γ-secretase complex, promoting the production of Aβ (Zhang X. et al., 2014). In AD patients, the highly expressed PSEN-1 is phosphorylated at serine 367 site, regulating the Aβ degradation function of microglia cells, thereby reducing the levels of Aβ (Ledo et al., 2021). The latest research shows that PSEN-1 can regulate the maturation and transport of acetylcholinesterase in the Golgi apparatus region, and affect the glycosylation of acetylcholinesterase. Mutations in PSEN-1 lead to decreased activity and altered glycosylation of acetylcholinesterase, affecting the extent of cholinergic damage in AD patients (Cortés-Gómez et al., 2023). NCSTN is responsible for substrate recognition in the γ-secretase complex, regulating the activity of the γ-secretase complex through interactions with its various subunits (Zhang X. et al., 2014). NCSTN overexpression can promote the growth and migration of liver cancer cells *in vitro* and *in vivo*. NCSTN activates the Notch1 and AKT signaling pathways, inhibiting the activity of GSK-3β. GSK-3β is a negative regulator of β-catenin, and when its activity is reduced, it inhibits the phosphorylation and degradation of β-catenin, leading to its accumulation. β-catenin enters the cell nucleus to regulate gene expression, promoting the growth of hepatocellular carcinoma. NCSTN can also cause nuclear translocation of β-catenin, initiating the transcription of the Zeb1 (transcription factor), leading to a malignant phenotype in hepatocellular carcinoma (Li H. et al., 2020). The function of APH-1 is to stabilize the structure of γ-secretase complex, regulate the activity of γ-secretase, and participate in the generation and metabolism of APP (Qin et al., 2011). PEN-2 interacts with the transmembrane-4 domain of presenilin to promote the formation and stability of the γ-secretase complex (Zhang X. et al., 2014). In addition, in the central nervous system, PEN-2 negatively regulates the differentiation of oligodendrocyte precursor cells into astrocytes. When PEN-2 is low-expressed, oligodendrocyte precursor cells are more likely to differentiate into astrocytes, mainly affecting the formation of the blood-brain barrier and inflammatory responses (Hou et al., 2021).

δ-secretase (AEP) plays a role in protein degradation, tumor development, inflammation regulation, and neurodegenerative diseases. δ-secretase selectively cleaves specific sites on proteins, promoting the degradation of aged, abnormal, or damaged proteins within cells to maintain protein homeostasis. Research has shown that δ-secretase can cleave proteins at the C-terminal of asparagine or glutamine residues (Fottner et al., 2022). Similarly, δ-secretase helps regulate the expression of tumor-related genes, and is involved in the proliferation, invasion, and metastasis of tumor cells. For example, high expression of δ-secretase can promote the progression of cancers such as breast cancer, glioblastoma, gastric cancer, and ovarian epithelial cancer (Zhang and Lin, 2021). In addition, δ-secretase can cleave and activate inflammation-related proteins, such as cytokines and inflammatory mediators, regulating the intensity and duration of inflammatory responses. Studies have found that in monocytes of healthy individuals, δ-secretase can increase the expression of anti-inflammatory cytokines (IL-10) while reducing the expression of pro-inflammatory cytokines (MCP-1), promoting the anti-inflammatory effects of macrophages (Lunde et al., 2020). δ-secretase is involved in the damage and death process of neurons, closely related to the development of diseases such as Alzheimer’s disease. It has been reported that δ-secretase cleaves Tau protein at residues N255 and N368 sites, promoting Tau hyperphosphorylation and aggregation, leading to neuronal toxicity and causing cognitive impairment in AD patients (Kang et al., 2020).

The biological function of ε-secretase is still under further research, but it has been found that the function of ε-secretase is relatively limited at present. ε-secretase can regulate the cleavage of membrane proteins inside cells, such as APP (St George-Hyslop and Fraser, 2012). The intracellular domain fragments generated by ε-secretase cleaving APP can regulate the tumor suppressor gene p53. The C-terminal fragment AICD-C50 derived from ε-secretase increases the activity and mRNA expression of p53 (Alves da Costa et al., 2006; Checler et al., 2007). In addition, ε-secretase plays an important role in regulating cell signaling, cell apoptosis, and synaptic plasticity. ε-secretase cleaves APP to release intracellular fragments and transmits information to the cell nucleus. This cleavage process is a key factor in nuclear receptor-mediated signal transduction and gene expression. Gene expression induced by specific environmental signals mediates neurons’ response to environmental changes, affecting neuron survival and synaptic function (Robakis, 2003).

The η-secretase (membrane-type matrix metalloproteinase 5) can promote neuroinflammation, affect neuronal excitability, and regulate the function of neural stem cells. η-secretase can enhance the pro-inflammatory effects of interleukin-1β in neuroinflammation. Interleukin-1β is highly expressed in the bodies of AD patients, and it induces neuroinflammation by activating the inflammasome (NLRP3) (Pilat et al., 2022). η-secretase binds to AMPA receptor binding protein and interacts with glutamate receptor interacting protein, promoting the localization of AMPA receptors to the cell membrane, enhancing the excitability of neurons (Monea et al., 2006). η-secretase regulates the function of adult neural stem cells by cleaving N-cadherin. N-cadherin is a cell adhesion protein that plays a role in maintaining the quiescent state of neural stem cells. Cleavage of N-cadherin by η-secretase in the extracellular membrane region leads to changes in the function of N-cadherin. These changes include weakened cell adhesion and cell detachment. Weakened cell adhesion enhances the proliferative capacity of neural stem cells, while increased cell detachment enhances the migratory ability of neural stem cells (Porlan et al., 2014).

## 3 Key substrates for secretase

### 3.1 Secretase cleaves Notch receptors

**FIGURE 3:**
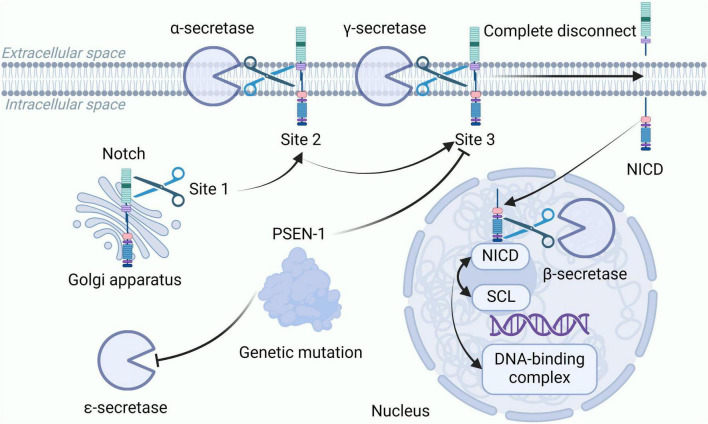
α, β, γ, and ε-secretases all play important roles in the activation of the Notch pathway. Notch is first cleaved at the S1 site in the Golgi, α-secretase is the second step hydrolase in the Notch pathway, β-secretase is responsible for cleaving NICD, which enters the nucleus and binds to the transcription factor CSL to form a DNA-binding complex, and γ-secretase performs S3 cleavage of the Notch receptor fragment. Mutations in the PSEN-1 gene inhibit ε-secretase cleavage and S3 cleavage of Notch. NICD, Notch intracellular domain; PSEN-1, presenilins-1.

The Notch signaling pathway is a highly conserved intercellular signaling system that regulates cell proliferation, apoptosis, differentiation, and survival, and is closely related to the occurrence and development of cancer (Krishna et al., 2019). Different subtypes of secreted enzymes play important roles in the activation of the Notch pathway, as shown in Figure 3. α-secretase belongs to the ADAM family. Among this family, ADAM10 is the most important α-secretase, with a cleavage activity accounting for 79–90% (Miranda et al., 2021). ADAM10 is the second step protease in the Notch pathway, playing an indispensable role in activating the Notch signaling pathway (Yuan et al., 2022). The β-secretase is responsible for cleaving the NICD, allowing NICD to be released into the nucleus, where it binds to the transcription factor CSL in the Notch signaling pathway, forming a DNA-binding complex. This complex can activate Notch target genes (Taylor et al., 2022). The γ-secretase cleaves the Notch receptor fragment at the third site (S3), releasing NICD into the cytoplasm, playing a crucial role in the activation and nuclear translocation of NICD (Das et al., 2019). The ε-secretase is also related to the S3 cleavage of Notch. Studies have shown that mutations in the PSEN-1 gene can inhibit the cleavage of ε-secretase near APP and the S3 cleavage of Notch. Beyreuther and colleagues referred to the cleavage at residues 49–52 of APP as ε-cleavage, and demonstrated that the ε-cleavage at residue 50 of APP and the S3 cleavage of Notch both depend on Presenilin (Chen et al., 2002). Currently, there are no reports on the relevance of δ-secretase and η-secretase to the Notch signaling pathway.

### 3.2 Secretase cleaves amyloid precursor protein (APP)

APP is a common substrate for various subtypes of secretory enzymes. ADAM10 is the main α-secretase involved in the processing of APP. When studying the effects of ADAM10 overexpression, gene knockout, or mutations on the levels of cleavage products and plaque formation, a large amount of evidence suggests that α-secretase plays a dominant role in the processing of APP (Xu et al., 2009). The dominant role of α-secretase in cleaving APP may be based on two reasons: a higher distribution of ADAM10 inside the cell can be observed under a microscope; ADAM10 is located closer to its substrate APP inside the cell (Hitschler and Lang, 2022). APP is a single-pass transmembrane protein that is cleaved by α-secretase in the middle region of Aβ, releasing a larger extracellular domain (APPsα) and leaving behind an 83-amino acid C-terminal fragment (APP-C83) in the membrane. Proteolytic cleavage of APP-C83 by γ-secretase can generate a peptide P3, which is a N-terminal truncated form similar to Aβ. Proteolytic cleavage of APP by β-secretase and γ-secretase can also produce Aβ, with γ-secretase having different cleavage sites, mainly cleaving after the 40th amino acid of Aβ, but also after the 42nd amino acid. Specifically, APP is first cleaved by β-secretase in the extracellular domain, producing a membrane-bound fragment containing 99 residues (CTF-β or APP-C99). APP-C99 is further cleaved by γ-secretase to release Aβ and NICD (Xie et al., 2005).

γ-secretase can bind to its substrates APP-C99, Aβ49, Aβ46, and Aβ43, respectively, revealing the mechanism of substrate recognition and cleavage. there is little difference between the endopeptidase and carboxypeptidase activities of γ-secretase, with the main difference being in the length of the β-strand formed by the substrate: for the production of Aβ49, C99 forms a relatively long β-strand region; for carboxypeptidase cleavage (Aβ49→Aβ46→Aβ43→Aβ40), the substrate β-strand region contains only three consecutive amino acid residues. As the substrate is shifted to the inner side of the cell for cleavage, the transmembrane α-helix region of the substrate maintains its overall length, and after each cleavage, the α-helix region of the substrate screws forward and translocate by one helix (about three amino acids) and forms a new β-strand, a mechanism summarized as the “piston model.” The substrates were found to share the same structural features: a transmembrane α-helix, a three-amino-acid residue linker peptide cleaved by γ-secretase, and a heterotrimeric β-folded strand formed with the PS1 protein. Protease hydrolytic cleavage occurs in front of the substrate β-folded chain. After each cleavage step, the substrate α-helix undergoes a deconvolution and translocation and forms a new β-folded chain. The selectivity of the cleavage site involves the three-dimensional structure of the substrate protein, the amino acid sequence, and the interaction with the γ-secretase enzyme. γ-secretase cleavage sites are usually located within the transmembrane region of the protein, a region that is relatively highly hydrophobic. This is because the transmembrane region usually consists of hydrophobic amino acid residues that contribute to the anchoring of the protein in the cell membrane. The substrate specificity of γ-secretase involves recognizing and binding to these hydrophobic transmembrane regions and cleaving at specific sites. For example, during the processing of APP, γ-secretase cleaves within the transmembrane region of APP to produce β-amyloid Aβ (Yang et al., 2020).

**FIGURE 4:**
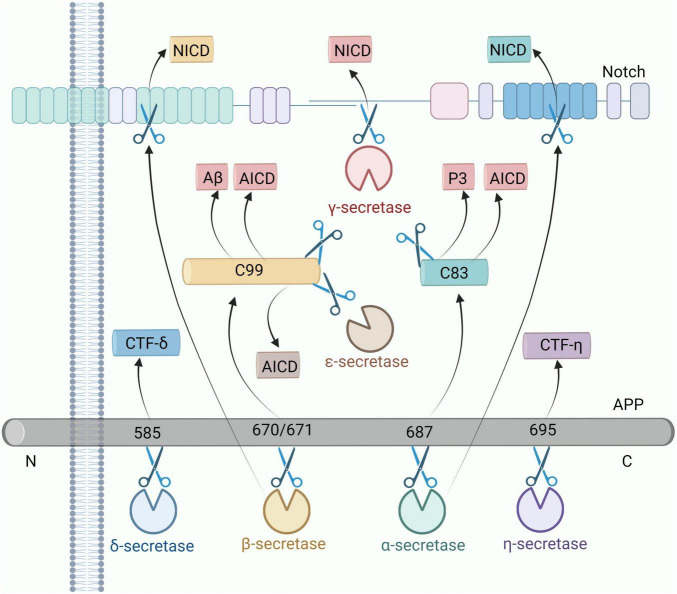
Each subtype of secretase cleaves APP and Notch as substrates. α-secretase shears APP at position 687 to produce the C83 fragment, and γ-secretase cleaves the C83 fragment to generate the peptide P3 and the AICD fragment. β-secretase cleaves APP at positions 670 methionine and 671 aspartic acids to produce the C99 fragment, and γ-secretase cleaves the C99 fragment to generate Aβ and the AICD fragment. δ-secretase cleaves C99 to produce CTF-δ, and ε-secretase cleaves C99 to generate the AICD fragment. AICD fragments, and ε-secretase cleavage of the C99 fragment also produces AICD fragments. δ-secretase shearing of the N585 site of APP produces CTF-δ fragments. η-secretase shearing of the 695 fragment of APP produces CTF-η fragments. In addition, α, β, and γ-secretase can cleave the Notch receptor to produce NICD fragment, respectively. AICD, APP intracellular domain; NICD, Notch intracellular domain; CTF-η, carboxy-terminal fragment-η; CTF-δ, carboxy-terminal fragment-δ; Aβ, amyloid-beta; APP, amyloid precursor protein.

AEP is a δ-secretase that cleaves APP in the brains of mice and AD patients (Zhang Z. et al., 2014). AEP cleaves APP after residues N373 and N585 in the extracellular domain leading to the generation of the APP C586-695 fragment (CTF-δ C110) and the production of Aβ (Yao et al., 2021). ε-secretase also cleaves APP. ε-secretase can cleave β-APP between residues 49 and 50 in the Aβ domain, as well as between residues 48 and 49. In addition, APP-ε is an N-terminal fragment derived from ε-secretase in APP, which is membrane-bound and targeted for cleavage by α, β, and γ-secretases (Lefranc-Jullien et al., 2006). The latest research has found that membrane-type matrix metalloproteinase 5 is a novel APP-cleaving enzyme, also known as η-secretase. The APP fragment CTF-η derived from η-secretase is localized in the Golgi apparatus, the nucleus, and extracellular vesicles. CTF-η undergoes cleavage by α-secretase and β-secretase to produce Aη-α and Aη-β peptides, which contribute to the production of Aβ (Afram et al., 2023). The cleavage of APP and Notch by various subtypes of secretase is shown in Figure 4.

### 3.3 Secretase regulates voltage-gated channels

Voltage-gated sodium channels (VGSC) are heteromeric complexes composed of Na+ conducting α subunits and non-pore-forming β subunits. VGSC is primarily expressed in excitable cells, such as neurons. VGSC regulates Na+ influx, generates action potentials, and conducts excitability. VGSC is also present in non-excitable cells, such as cancer cells. VGSC can influence a range of biological functions, such as phagocytosis and altering enzyme activity (Black and Waxman, 2013). Cleavage of secretase is of great significance to the gated channel. VGSC is the first ion channel identified as a target of β-secretase (Wong et al., 2005). α, β, and γ-secretases can all cleave the β1 subunit of VGSC, releasing the extracellular domain and intracellular domain, respectively. The β1 subunit can regulate cell surface expression and gating of the α-subunit, and is involved in cell adhesion. Subsequently, cleavage sites of β-secretases were found on the β2 subunit, and the proteolysis of β2 by β-secretases can lead to a voltage-dependent left shift in VGSC (Haworth et al., 2022). Research shows that VGSC is related to the invasion and metastasis of cancer cells, and it is abnormally expressed in various types of cancers, such as BC (Angus and Ruben, 2019). The Nav1 family of VGSC includes 9 genes (SCN1A-5A, SCN8A-11A), among which Nav1.5 is the pore-forming α subunit encoded by SCN-5A. Nav1.5 is highly expressed in metastatic BC cells. Abnormal activation and expression of Nav1.5 can trigger various cellular mechanisms, including altering H+ efflux, promoting epithelial-mesenchymal transition, and enhancing the expression of cysteine proteases, to increase the invasive and metastatic abilities of BC cells *in vitro* and *in vivo* (Luo et al., 2020). NaV1.5 is highly expressed in colorectal cancer cell lines, and the electrical conductivity of NaV1.5 can significantly promote the invasion and progression of colorectal cancer cells (Anderson et al., 2019).

**FIGURE 5:**
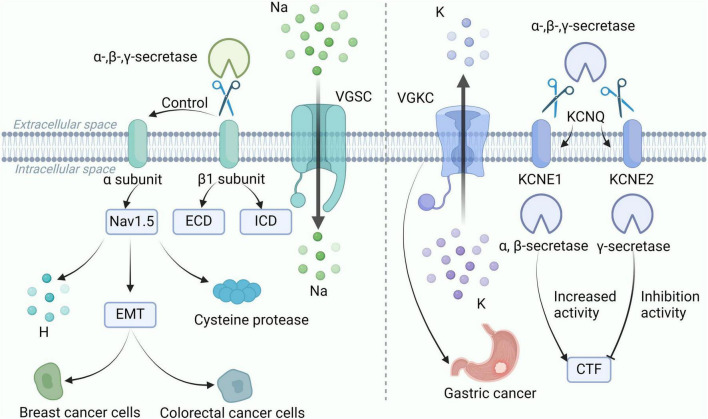
α, β, and γ-secretases are closely associated with VGSC and VGKC channels. α, β, and γ-secretase shear the β1 subunit of VGSC to release ECD and ICD. The β1 subunit regulates the gating of the α-subunit, and the α-subunit Nav1.5 alters H+ efflux and promotes the expression of EMT and cysteine histone protease to enhance expression in breast and colorectal cancer cells. In addition, α, β and γ-secretase shear the KCNE1 and KCNE2 subunits of KCNQ, and VGKC is associated with gastric cancer. elevated α-secretase or β-secretase activity increased the CTF level of the KCNQ subunit, whereas inhibition of γ-secretase activity increased the CTF level. VGSC, voltage-gated sodium channels; VGKC, voltage-gated potassium channel; CTF, carboxy-terminal fragment; ECD, extracellular domain; ICD, intracellular domain; EMT, epithelial-mesenchymal transition.

In addition, the secretion of enzymes also has a regulatory effect on the voltage-gated potassium channel (VGKC). The VGKC channel family has five members, namely KCNQ1-5 (Lehnert et al., 2016). This channel can determine a series of physiological responses, including the frequency and duration of action potential discharges, muscle contraction, and hormone secretion. The opening of the K+ channel leads to K+ efflux, causing a stable hyperpolarization of the cell membrane potential, which inhibits cell excitability (Barrese et al., 2018). It has been shown that secretase regulates KCNQ and assists in the processing of the β-subunit, thereby modulating membrane excitability. The KCNQ subunits (KCNE1 and KCNE2) undergo sequential α/β/γ-secretase-mediated cleavage in the cell. Elevated α-secretase or β-secretase activity increases CTF levels of the KCNQ subunit and shifts the activation curve of the KCNE1/KCNQ1 channel. Inhibition of γ-secretase activity increases endogenous KCNE1-CTF and KCNE2-CTF levels 2–4 fold (Sachse et al., 2013). The cleavage of KCNE1 and KCNE2 under physiological conditions is regulated by secretase activity, and that KCNE cleavage plays a functional role in the regulation of KCNQ. Secretases can affect VGKC channels through protein hydrolysis and shearing. Recent studies have shown that KCNQ plays an important role in the control of membrane potential and intestinal ion homeostasis, and it is linked to gastrointestinal cancers. A study analyzing a large sample of 897 patients with gastroesophageal adenocarcinomas and incorporating *in vitro* models found that the KCNQ family of genes is mutated in 30% of patients and plays a role in therapeutic targeting for inhibiting gastroesophageal adenocarcinomas growth (Shorthouse et al., 2023). The above shows that α, β, and γ-secretase are closely related to ion channels, and secretase can be indirectly associated with cancer through ion channels, as shown in Figure 5.

## 4 The secretase cleaves Notch receptor to increase NICD accumulation and promote the progression of cancer

α-secretase is closely related to BC. It was found that α-secretase can be overexpressed in different types of BC cell lines, and the fragments hydrolyzed by α-secretase from the substrate can be detected (Tsang et al., 2018). It has been shown that the shedding of the extracellular domain of human epidermal growth factor receptor 2 in BC cells is associated with the expression of α-secretase (represented by ADAM10) and the prognosis of BC. Inhibition of α-secretase activity can reduce extracellular domain shedding and effectively improve the prognosis of BC (Zheng et al., 2019). The Notch pathway plays a major role in BC progression and drug resistance therapy, and α-secretase is the second-step cutting enzyme activated by the Notch receptor (Chimento et al., 2022). α-secretase cleaves the Notch receptor, releasing the Notch extracellular domain, which is subsequently cleaved by γ-secretase, releasing NICD. NICD translocate from the cytoplasm to the nucleus and binds to the transcription factor CSL, recruiting a family of transcription-activating proteins, initiating gene transcription and promoting BC tumor growth (BeLow and Osipo, 2020).

γ-secretase correlates more closely with BC. γ-secretase is a crucial protein cleavage enzyme in the Notch pathway to turn on transcription of downstream genes (Jia et al., 2021a). γ-secretase inhibitors (GSIs) block the Notch pathway activation and exert anti-BC effects (Means-Powell et al., 2022). It has been shown that Cimigenoside, a naturally occurring GSIs, improves BC migration and invasion by inhibiting the Notch pathway-mediated epithelial-mesenchymal transition, and it is expected to be developed as a targeted precursor drug for BC treatment (Jia et al., 2021a). NCSTN is the subunit of the γ-secretase complex responsible for recognizing the active site, and NCSTN regulates BC progression *in vitro* and *in vivo* by mediating the growth of BC stem cells through the Notch1 and AKT (protein kinase B) pathways. It was shown that stable knockdown of NCSTN in HCC1806 BC cells would result in a 51.4 ± 1.7% reduction in cell invasion and a 2–3 fold reduction in BC stem cells (Lombardo et al., 2012). δ-secretase has an impact on the prognosis of BC patients, and effective inhibition of δ-secretase activity can block BC cell invasion and metastasis, therefore, δ-secretase is expected to be developed as a biomarker for BC treatment (Gawenda et al., 2007; Lin et al., 2014). Another report δ-secretase inhibitor (BIC-133) combined with epirubicin can reduce the number of osteoclasts and effectively prevent BC bone metastasis (Chen et al., 2023).

Various subtypes of secretases are strongly associated with different types of cancer. ADAM9/10/17 and other members of the ADAM family, as typical α-secretase, are known to promote the metastasis and invasion of pancreatic ductal adenocarcinoma by enhancing their activity (Lu W. et al., 2020). In addition, α-secretase has been associated with glioblastoma. It has been shown that activation of the Notch pathway promotes the development of glioblastoma, and α-secretase inhibitors inhibit the growth of glioblastoma by targeting ADAM10/17 and inhibiting the activation of the Notch pathway (Floyd et al., 2012).

β-secretase was associated with gastric cancer, glioma, and ocular melanoma, and its effect on gastric cancer and the other two cancers was reversed. β-secretase was significantly down-regulated in gastric cancer samples but highly expressed in gliomas and melanomas. The expression of β-secretase was significantly lower in gastric cardia tumor samples than in other tumor samples, indicating that the expression level of β-secretase was related to the site of the primary tumor (Esfandi et al., 2019). In contrast, the overexpression of β-secretase can promote the rapid proliferation of glioma cells, while the decreased activity of β-secretase (BACE2) can effectively inhibit the growth of glioma (Wang et al., 2020b). Similarly, β-secretase is significantly upregulated in ocular melanoma, and inhibiting β-secretase *in vivo* and *in vitro* can effectively treat ocular melanoma (He et al., 2021).

γ-secretase also plays an important role in the treatment of hepatocellular carcinoma, aggressive fibroma, sarcoma, and adenoid cystic carcinoma. Some studies have found that overexpression of NCSTN can promote the proliferation of hepatocellular carcinoma *in vitro*, and down-expression of NCSTN can inhibit the growth of hepatocellular carcinoma (Jia et al., 2021b). Nirogacesta (a GSIs) exhibits anti-aggressive fibroma activity by inhibiting the activation of the NICD/Notch axis (Gounder et al., 2023). RO4929097 (a GSIs) targeting γ-secretase inhibits the growth of multiple sarcomas, promotes tumor cell apoptosis, and increases chemotherapy sensitivity (Gounder et al., 2022). Adenoid cystic carcinoma is an aggressive salivary gland malignancy. AL101 (a GSIs) has good specificity for the Notch signaling pathway, and it can effectively inhibit Notch mutant adenoid cystic carcinoma progression (Ferrarotto et al., 2022).

δ-secretase is associated with gastric cancer, glioblastoma, ovarian cancer, rectal cancer, and prostate cancer. In tumor tissue samples, δ-secretase is highly expressed in gastric cancer, which affects the prognosis of patients (Li et al., 2013). Reducing the expression of δ-secretase in tumor-associated macrophages can inhibit the progression of gastric cancer (Wang et al., 2020a). In glioblastoma cells (U87-MG and A172 cell lines), δ-secretase activity is increased, and the N311 site of p53 is cleaved, resulting in loss of p53 tumor suppressor function. In a mouse model, inhibition of δ-secretase activity reduced glioblastoma progression and prolonged the survival of mice (Lin et al., 2020; Manfredi, 2020). δ-secretase is highly expressed in both ovarian cancer cells and peritoneal mesothelial cells, and δ-secretase can promote peritoneal metastasis of ovarian cancer (Zhang and Lin, 2021). In patients with rectal cancer, patients with high expression of δ-secretase have shorter overall survival, indicating that δ-secretase may be involved in the progression of rectal cancer (Haugen et al., 2013; Zhen et al., 2015). Elevated δ-secretase activity was also detected in prostate cancer cells (PC-3, DU-145, LNCaP, and C4-2) (Stern et al., 2009; Ohno et al., 2013). Many studies have confirmed that δ-secretase is highly expressed in human solid tumors, and reducing δ-secretase activity will help to slow down tumor growth. The correlation between ε-secretase and η-secretase and cancer has not been reported.

## 5 The secretase cleaves APP to increase Aβ accumulation and promote the progression of AD

**FIGURE 6:**
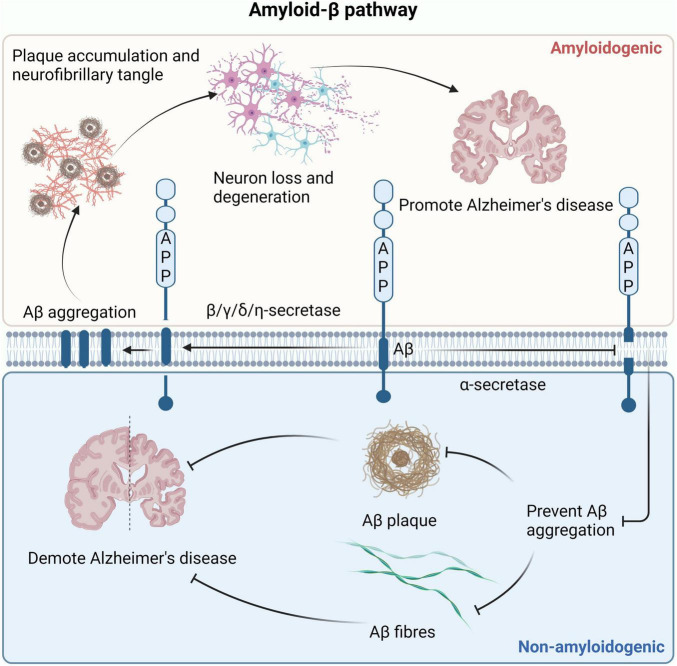
Two pathways that affect Aβ production. In the amyloid production pathway, β, γ, δ, and η-secretase can cleave APP to produce Aβ. The accumulation of Aβ leads to the formation of amyloid fibrils and plaques, causing neuronal cell death and ultimately promoting the development of AD. In the non-amyloid protein production pathway, α-secretase clears APP to prevent the formation of Aβ, eliminating the possibility of amyloid plaque formation and delaying the development of AD. AD, Alzheimer’s disease; APP, amyloid precursor protein.

AD is a neurological degenerative disease, the most common pathological sign is Aβ peptides, which leads to the deposition of extracellular amyloid plaques due to synaptic and neuronal damage (Dai and Shen, 2021). There are two distinct paths affecting Aβ production, as shown in Figure 6. In the amyloid pathway, secretase is a factor conducive to the production of Aβ. β, γ, δ, η-secretase can cleave AAP, promote Aβ production, and affect the progression of AD (Lichtenthaler et al., 2022). It has been found that inhibition of Aβ formation can slow the progression of AD in β-secretase deficient mice (Ghosh and Osswald, 2014). High expression of γ-secretase leads to high susceptibility to AD. By reducing the expression of γ-secretase, the production of Aβ in AD mouse models was reduced, and the symptoms of AD were alleviated (Jin et al., 2022). High expression of δ-secretase and increased Aβ accumulation can be detected in AD patient samples (Chen C. et al., 2021). Increased activity of η-secretase leads to excessive production of Aβ, which causes abnormal aggregation and deposition of Aβ, forming age spots and nerve fiber tangles, and impacting neuronal function (Mensch et al., 2021). Recent studies have found that gene knockout of PCSK6 (a serine protease) can inactivate η-secretase and effectively improve cognitive impairment in AD (Xu et al., 2024). In contrast, in the non-amyloid pathway, secretase is the factor that inhibits the production of Aβ. α-secretase clears the middle region of APP, prevents the formation of Aβ, eliminates the possibility of amyloid plaque formation, and delays AD development (Imbimbo et al., 2023). ε-secretase does not belong to one of the two pathways, but it is also related to AD. ε-secretase produces AICD by shearing the C terminus of APP, and the abnormal accumulation of AICD has been implicated in the pathogenesis of AD (Ward et al., 2010).

## 6 Development of secretase inhibitors for various subtypes

In recent years, various subtypes of secretase inhibitors have played a crucial role in the field of cancer and AD therapy, as shown in Table 2. Secretase inhibitors inhibit enzyme catalysis through binding to the active site of the secretase and preventing substrate interaction with the enzyme (Nie et al., 2020). At present, a number of α-secretase inhibitors have been developed, such as TPI-1, LT4, CAM29, MN8, SN-4, and GI254023X. Studies have shown that ADAM17 inhibitors can promote the proliferation and differentiation of natural killer cells, improve the killing effect of natural killer cells by activating the immune system, and increase the antibody-dependent cytotoxicity of natural killer cells to BC cells, which is expected to be developed as a BC treatment (2017). In Hodgkin’s lymphoma cells, LT4, CAM29, and MN8 act to reduce the release of ADAM10 substrates (CD30 and TNF-α), lower the ATP content of lymphoma cells, and increase the release of lactate dehydrogenase, ultimately inhibiting the growth and proliferation of lymphoma cells (Pece et al., 2022). Among them, LT4 and CAM29 inhibited the release of CD30, which enhanced the biological effect of the anti-tumor agent Brentuximab Vedotin on Hodgkin lymphoma cells, and interfered with exosome exchange between Hodgkin lymphoma cells and lymph node stromal cells, thereby affecting the activity of α-secretase (Tosetti et al., 2018). SN-4 is a potential anti-metastasis agent. SN-4 is able to inhibit ADAM17-mediated cleavage of proteins in the extracellular region, such as TNF-α and CD44, whose cleavage correlates with the metastatic and invasive ability of cells (Tateishi et al., 2021). By inhibiting the activity of α-secretase, GI254023X weakens intercellular adhesion and signaling, thereby inhibiting tumor cell invasion and metastasis. In 3D cell culture, GI254023X interferes with the integration of the spheroid structure of glioma stem cells into the organoid structure, affecting tissue repair and regeneration (Goranci-Buzhala et al., 2020).

**TABLE 2:** List of secretase inhibitors.

Inhibitors subtype	Agent	Chemical structure	Indication	Study stage
α-secretase inhibitors	TAPI-1	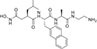	Breast cancer, pancreas, liver cancer, esophageal squamous cell carcinoma (Gao et al., 2024), rheumatoid arthritis, renal injury, neuroinflammation (Sultana and Bishayi, 2020)	Preclinical studies (*in vitro* and *in vivo* experiments)
	LT4	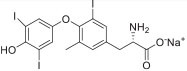	Hodgkin lymphoma (Smith et al., 2020; Pece et al., 2022)	Preclinical studies (*in vitro* experiments)
	SN-4	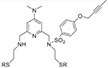	Diabetic (Toma et al., 2024)	Preclinical studies (*in vitro* experiments)
	GI254023X	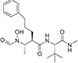	Breast cancer, glioblastoma (Smith et al., 2020)	Phase I
	INCB7839	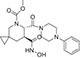	Breast cancer, glioblastoma, neuroglioma (Smith et al., 2020)	Phase II
	INCB3619	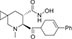	Non-small cell lung cancer (Zhou et al., 2006), breast cancer (Witters et al., 2008), colon cancer (Mustafi et al., 2017)	Preclinical studies (*in vitro* and *in vivo* experiments)
β-secretase inhibitors	PF-06751979	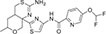	Alzheimer’s disease (O’Neill et al., 2018)	Stop-stage I
	LY-2811376	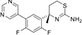	Alzheimer’s disease (May et al., 2011)	Stop-stage I
	LY-2886721	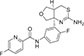	Early-onset Alzheimer’s disease (Li L. et al., 2020)	Stop-stage II
	JNJ-54861911	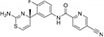	Alzheimer’s disease (Monteiro et al., 2023)	Stop-stage II/III
	CNP-520	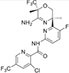	Alzheimer’s disease (Rombouts et al., 2021)	Stop-stage II/III
	E-2609	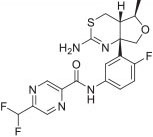	Mild Alzheimer’s disease (Rombouts et al., 2021; Patel et al., 2022)	Stop-stage III
	AZD-3293/LY3314814	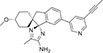	Alzheimer’s disease (Rombouts et al., 2021; Patel et al., 2022)	Stop-stage III
	MK-8931	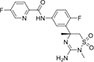	Glioblastoma (Zhai et al., 2021), Alzheimer’s disease (Rombouts et al., 2021)	Stop-stage III
γ-secretase inhibitors	Fosciclopirox (CPX-POM)	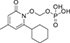	Urothelial cancer (Weir et al., 2021)	Phase II
	Abemaciclib mesylate	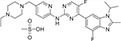	Alzheimer’s disease (Lee and Hoe, 2023)	Marketed drug (Eli Lilly and Company, Verzenio)
	LY411575	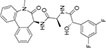	Alzheimer’s disease (Jia et al., 2021b), triple-negative breast cancer (Sen and Ghosh, 2023), liver cancer (Kasai et al., 2021), oral cancer (Ghosh et al., 2023), rheumatoid arthritis (Chen J. et al., 2021), lung adenocarcinoma (Tong et al., 2021)	Preclinical studies (*in vitro* and *in vivo* experiments)
	YO-01027 (DBZ,Dibenzazepine)	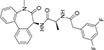	Colitis (Ahmed et al., 2023), tubulointerstitial nephritis (Tang et al., 2020), colorectal cancer (Usui et al., 2018)	Preclinical studies (*in vitro* and *in vivo* experiments)
	RO4929097 (RG-4733)	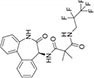	Oral cancer (Ghosh et al., 2023), glioblastoma (Peereboom et al., 2021), multiple myeloma (Pont et al., 2019), melanoma (Thippu Jayaprakash et al., 2020), breast cancer (Means-Powell et al., 2022), prostate cancer (Du et al., 2021), pancreatic ductal adenocarcinoma (Sheng et al., 2022), lung adenocarcinoma (Lin et al., 2022), renal cell carcinoma (Han et al., 2020), osteosarcoma (Yang et al., 2021), cervical cancer (Low et al., 2020)	Phase II
	PSEN1-IN-2 (Compound13K)	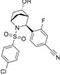	Alzheimer’s disease (Narlawar et al., 2023)	Preclinical studies (*in vitro* and *in vivo* experiments)
	PSEN1-IN-1 [Compound (+)-13b]	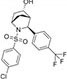	Alzheimer’s disease (Narlawar et al., 2023)	Preclinical studies (*in vitro* and *in vivo* experiments)
	BMS-299897	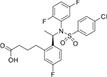	Alzheimer’s disease (Kounnas et al., 2010)	Preclinical studies (*in vitro* and *in vivo* experiments)
	ELN-318463	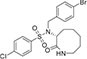	Alzheimer’s disease (Basi et al., 2010)	Preclinical studies (*in vitro* and *in vivo* experiments)
	BMS-433796	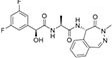	Alzheimer’s disease (Prasad et al., 2007)	Preclinical studies (*in vitro* and *in vivo* experiments)
	Sulindac sulfide	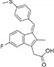	Colorectal cancer (Horinaka et al., 2014), Alzheimer’s disease (Prade et al., 2016), thyroid cancer (Hong et al., 2023), triple-negative breast cancer (Soundararajan et al., 2009)	Preclinical studies (*in vitro* and *in vivo* experiments)
	MRK-560	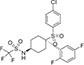	Alzheimer’s disease (Guo et al., 2022), acute lymphoblastic leukemia (Govaerts et al., 2021)	Preclinical studies (*in vitro* and *in vivo* experiments)
	NGP-555	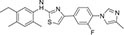	Alzheimer’s disease (Jia et al., 2021b)	Phase I
	MK-0752	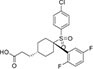	Ovarian cancer (Chen et al., 2016), colorectal cancer (Brana et al., 2014), pancreatic ductal adenocarcinoma (Cook et al., 2018), breast cancer (Wang et al., 2017), lung adenocarcinoma (Jiang et al., 2021), glioma (Shen et al., 2016), melanoma (Su et al., 2021), head and neck squamous cell carcinoma (Varatanovic et al., 2023), renal injury (Ghafil et al., 2023), acute lymphoblastic leukemia (Greene et al., 2016)	Phase I
	E-2012	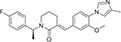	Alzheimer’s disease (Yesuvadian et al., 2014)	Phase I
	BMS-906024	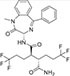	Lung adenocarcinoma (Morgan et al., 2017), acute lymphoblastic leukemia (Boisson and Casanova, 2021), adenoid cystic carcinoma (Wang et al., 2022), non-small cell lung cancer (Sosa Iglesias et al., 2018), Alzheimer’s disease (Jia et al., 2021b)	Preclinical studies (*in vitro* and *in vivo* experiments)
	Crenigacestat (LY3039478)	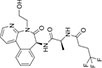	Intrahepatic cholangiocarcinoma (Mancarella et al., 2020), T-cell acute lymphoblastic leukemia, T-cell lymphoblastic lymphoma (Borthakur et al., 2021), multiple myeloma (García-Guerrero et al., 2023),soft tissue sarcoma (Carvalho et al., 2014), adenoid cystic carcinoma (Even et al., 2020), head and neck squamous cell carcinoma (Anameriç et al., 2023), diabetic (Lu et al., 2021), osteosarcoma (Lu B. et al., 2020), gastric cancer (Wang et al., 2018)	Phase II
	Semagacestat (LY450139)	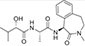	Alzheimer’s disease (Jia et al., 2021b)	Phase III
	Compound E	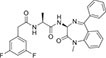	Alzheimer’s disease (Jia et al., 2021b)	Preclinical studies (*in vitro* experiments)
	Nirogacestat (PF-03084014)	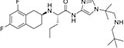	Desmoid tumors (Gounder et al., 2023), hypophosphatemia (Gudsoorkar et al., 2023), chronic lymphocytic leukemia (López-Guerra et al., 2015), liver cancer (Wu et al., 2017), prostate cancer (Cui et al., 2015), T-cell acute lymphoblastic leukemia, T-cell lymphoblastic lymphoma (Papayannidis et al., 2015), triple-negative breast cancer (Wang et al., 2015), multiple myeloma (Nooka et al., 2021), pancreas (Wang S. et al., 2021), melanoma (Keam, 2024), tracheal adenoid cystic carcinoma (Kieran et al., 2021)	Phase III, drugs marketed in the United States (desmoid tumors) (Keam, 2024)
	DAPT (LY374973)	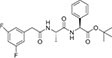	Alzheimer’s disease (Sogorb-Esteve et al., 2018)	Preclinical studies (*in vitro* and *in vivo* experiments)
	L-685458	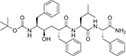	Lung cancer (Zhang et al., 2019)	Preclinical studies (*in vitro* and *in vivo* experiments)
	LY900009	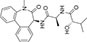	Ovarian cancer (Pant et al., 2016)	Phase I
	CHF5074 (CSP-1103)	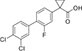	Alzheimer’s disease (Porrini et al., 2017)	Phase III
	BT-GSI	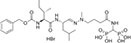	Multiple myeloma (Sabol et al., 2021)	Preclinical studies (*in vitro* and *in vivo* experiments)
	GSI-I(Z-LLNle-CHO)	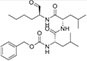	Prostate cancer (Mohamed et al., 2017), acute lymphoblastic leukemia (Meng et al., 2011), breast cancer (Han et al., 2009), alveolar rhabdomyosarcoma (Marshall et al., 2013)	Phase I
	NNC 26-9100	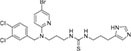	Alzheimer’s disease (Sandoval et al., 2019)	Preclinical studies (*in vitro* and *in vivo* experiments)
	Z-Phe-Ala-diazomethylketone	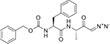	Alzheimer’s disease, Parkinson’s disease (Hwang et al., 2019)	Preclinical studies (*in vitro* and *in vivo* experiments)
δ-secretase inhibitors	Compound 11	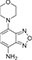	Alzheimer’s disease (Wang J. et al., 2021)	Preclinical studies (*in vitro* and *in vivo* experiments)

Between 2011 and 2020, researchers identified nearly 146 β-secretase inhibitors (Rombouts et al., 2021). Currently, the β-secretase inhibitors LY-3002813, LY-3372993, and JNJ-63733657 are in Phase III clinical studies, and LY3372689 is in phase II clinical studies (Monteiro et al., 2023). However, some β-secretase inhibitors have been terminated due to drug toxicity and lack of efficacy. MK-8931 is an oral drug developed by Merck. In mild-to-moderate AD patients aged 55 to 85 years, MK-8931 reduces Aβ40 and Aβ42 and inhibits β-secretase activity. But some adverse effects were observed, such as weight loss, rash, sleep disturbance, etc (Moussa-Pacha et al., 2020). LY3314814 is a drug developed by AstraZeneca and Eli Lilly. Despite promising results *in vitro*, the trial was stopped early in phase III without significant efficacy. In neuroimaging findings, the total load of Tau neurofibrillary tangles, perfusion, and brain metabolism did not change significantly at baseline. Although the treatment was well tolerated, the patient experienced cognitive decline (Zimmer et al., 2021). JNJ-54861911 has been developed and tested by Janssen to reduce the concentration of Aβ in cerebrospinal fluid. A six-month study was conducted in early-stage AD patients aged 50–85 years. The subjects showed elevated liver enzymes and decreased cognitive ability, and the trial was terminated early (Novak et al., 2020).

As a potential therapeutic target for cancer and AD, GSIs have attracted the attention of many scholars in the world (Feng et al., 2024; Jia et al., 2021b; Kumar et al., 2018; Santiago et al., 2021). The main functions of GSIs include inhibition of Notch signaling pathway and inhibition of APP metabolism. GSIs play an anti-tumor role by inhibiting the activity of γ-secretase and blocking the conduction of Notch signaling pathway, thereby inhibiting the proliferation and invasion ability of tumor cells. In addition, GSIs can inhibit the shearing effect of γ-secretase on APP, reduce the production of Aβ, and delay the progression of AD (Kumar et al., 2018). For example, LY-411575 and BMS-708163 reduce the production of Aβ, which is beneficial for the treatment of AD (Mouchlis et al., 2020). NMK-T-057 and PF-03084014 are potential candidates for anti-BC treatment, as they induce apoptosis by inhibiting γ-secretase mediated Notch signaling activation (Das et al., 2019; Hossain et al., 2018). A new study shows that an oral GSIs (Nirogacestat) has been approved by the US FDA for the first time for the treatment of fibroids. It inhibits Notch signaling mediated growth of desmoid cells by blocking γ-secretase activity (Kingwell, 2024). Semagacestat, a small molecule developed by Eli Lilly and Company, was the first GSI to enter Phase III clinical trials, and Avagacestat, developed by BMS, was the first GSI to enter Phase II clinical trials. Semagacestat and Avagacestat are both non-transition state analogs of GSIs. In addition, there are transition-state analogs of γ-secretase that bind to the structure of the TSA inhibitor L685,458. Many of the chemical tools used to study conformational changes in γ-secretase are derivatives of L685,458, and a portion of L685,458 has the same space occupation as the previous two inhibitors. L685,458 also interacts directly with the two aspartic acid catalytic residues of γ-secretase. L685,458 has a direct interaction with the two aspartic acid catalytic residues of γ-secretase. The structure of γ-secretase can bind to the structure of the TSA inhibitor L685,458 (Yang et al., 2020). Although GSIs have great application potential, they also have three disadvantages. Firstly, GSIs do not distinguish between Notch paralogues, which inhibit all Notch receptors. Some Notch receptors are tumor suppressor genes (Notch-2 receptors) whose expression should not be inhibited. Secondly, GSIs affect other targets outside the Notch signaling pathway, such as APP. Thirdly, taking GSIs is easy to induce gastrointestinal toxicity (Jia et al., 2021b). Based on these shortcomings, the researchers proposed the following solutions: (1) Optimizing GSIs design strategies, such as adjusting drug sequence length and altering target selection, to enhance the specific inhibition of Notch receptors; (2) The Notch pathway was affected by screening appropriate GSIs concentrations from Notch substrate inhibition profiles; (3) Intermittent dosing or preparation of GSIs into nanoparticles can reduce gastrointestinal side effects during treatment (Feng et al., 2024).

Existing studies of δ-secretase (AEP) inhibitors have targeted their antitumor effects. For example, matrix metalloproteinase 2 is involved in migration and invasion of human BC MDA-MB-231 and MDA-MB-435 cells. Compound 38u inhibits δ-secretase shearing of matrix metalloproteinase 2 in a dose-dependent manner and modulates matrix metalloproteinase 2 activity. Prolonged treatment of nude mice inoculated with MDA-MB-231 cells by oral administration of the inhibitor 38u significantly reduced or even completely prevented the occurrence of lung metastasis of BC. Moreover, no obvious toxic side effects of 38u were observed in mice, such as weight change and organ toxicity (Qi et al., 2017). Besides, ubiquitin-specific protease (USP17) inhibits BC development and progression by regulating the protein level of δ-secretase and interfering with the ERK signaling pathway (Chen et al., 2019). The correlation of ε, η- secretase inhibitors has not been reported, but the development of other subtypes of secretase inhibitors can indicate the direction.

## 7 Discussion

In the past twenty years, due to the crucial role of secretases in the pathogenesis of cancer and AD, we are in an exciting time of exploring secretase inhibitors. This article reviews six subtypes of secretases: α-secretase, β-secretase, γ-secretase, δ-secretase, ε-secretase, and η-secretase. The commonalities among these subtypes of secretases mainly include three points: (1) each subtype of secretase acts on APP as a substrate, but with different cleavage sites and products; (2) each subtype of secretase cleaves Notch receptors; (3) each subtype of secretase is closely related to the progression of cancer and AD, and secretase inhibitors play an important role in the treatment of cancer and AD. Additionally, the most significant difference among the subtypes of secretases lies in their distinct biological functions. In conclusion, by reviewing the commonalities and differences among the subtypes of secretases, this article provides a theoretical basis for the future development of secretase inhibitors, aiming to pave the way for new approaches to prevent or treat cancer and AD.

The main factors leading to the failure or slow development of drug therapy with secretase inhibitors are insufficient ion permeability, poor selectivity, significant side effects, or inappropriate targeting of therapeutic sites. Some secretase inhibitors need to cross the blood-brain barrier to take effect, but due to the large molecular weight and polarity of certain drugs, they cannot penetrate the blood-brain barrier, resulting in insufficient drug concentration in the brain and affecting treatment efficacy. Secretase inhibitors need to have high selectivity, for example, only acting on β-secretase without affecting other related substrates. Lack of selectivity can lead to adverse reactions or poor treatment outcomes. Secretase inhibitors can cause adverse reactions, such as skin toxicity or cognitive impairment in patients. Some secretase inhibitors primarily treat AD by inhibiting the production of Aβ. However, research has shown that Aβ is not the sole pathogenic factor of AD, and solely inhibiting Aβ may not completely cure or reverse AD (Monteiro et al., 2023). These issues need to be better addressed in future drug development.

In addition, chemotherapy resistance is a challenge in the field of cancer treatment. Poor clinical prognosis, metastasis, and recurrence are important factors leading to high cancer mortality rates, all of which are related to drug resistance. Therefore, reducing tumor drug resistance is key to improving the survival rate of cancer patients. For example, activation of the γ-secretase/Notch signaling pathway can promote drug resistance in breast cancer. GSIs can reverse tumor cell resistance to drugs, induce tumor cell apoptosis, and synergistically inhibit tumor cell growth with conventional chemotherapy drugs (Feng et al., 2024). The high correlation between various subtypes of secretases and the Notch pathway, with the Notch signaling pathway being a key in reversing tumor drug resistance. In the future, further exploration can be done on the therapeutic strategy of using secretase inhibitors to reverse tumor drug resistance. Combination therapy can also be attempted by using inhibitors of different subtypes of secretases together, leveraging the synergistic effects between different drugs to enhance treatment efficacy.

This article elaborates on the secretases with APP and Notch as the main cleavage substrates, indicating that different subtypes of secretases play a crucial role in AD and cancer progression. Various subtypes of secretases regulate Aβ levels by cleaving App. β, γ, δ, η-secretases cleave AAP, promoting the generation of Aβ and accelerating the progression of AD. The difference is that α-secretase inhibits the generation of Aβ by cleaving the middle region of APP, preventing the formation of Aβ, eliminating the formation of amyloid fibrils and plaques, and delaying the development of AD. Additionally, the secretase cleaves the Notch receptor to activate the Notch pathway, affecting the progression of breast cancer. For example, α-secretase is the second-step cleaving enzyme for Notch receptor activation, β-secretase is responsible for cleaving the intracellular segment of the Notch receptor, and γ-secretase cleaves the Notch receptor fragment at the third site. Interestingly, γ-secretase can cleave at least 149 substrates within its transmembrane domain (Güner and Lichtenthaler, 2020), which helps in the development of more effective secretase inhibitors.
